# Quinine Pills

**Published:** 1878-05

**Authors:** 


Quinia Pills.—As I have often seen formulas in various medical journals for compounding pills of quinia, none of which seem to have been satisfactory, permit me to inform you that if a small quantity of powdered gum arabic be added to the quinia and thoroughly mixed with it, and glycerin added, a few drops at a time, triturating well after each addition, it will make an excellent mass, which can be easily and leisurely worked into nice, smooth and compact pills, which will remain unalterable indefinitely.

I have used the above mentioned ingredients in the preparation of quina pills nearly seven years, and think if they are tried perfect satisfaction will result.—*Journal of Pharmacy.*

